# Correction: A microRNA-4516 inhibitor sensitizes chemo-resistant gastric cancer cells to chemotherapy by upregulating ING4

**DOI:** 10.1039/c8ra90089b

**Published:** 2018-11-22

**Authors:** Jun-bao Liu, Dan Chen, Hai-xia Liu, Huan-huan Sha, Dan Song, Ting-ting Bao, Jian-wei Lu, Chen Yu

**Affiliations:** Traditional Chinese Medicine Department, People’s Hospital of Henan Province, People’s Hospital of Zhengzhou University Zhengzhou Henan 450003 China; Research Center of Clinical Oncology, Jiangsu Cancer Hospital, Jiangsu Institute of Cancer Research & The Affiliated Cancer Hospital of Nanjing Medical University Nanjing Jiang Su 210000 China; Emergency Department, The Second Affiliated Hospital of Nanjing University of Chinese Medicine Nanjing Jiang Su 210017 China; The Forth Clinical School of Nanjing Medical University Nanjing Jiang Su 210000 China; Department of Radiotherapy, Jiangsu Cancer Hospital, Jiangsu Institute of Cancer Research & The Affiliated Cancer Hospital of Nanjing Medical University Nanjing Jiang Su 210000 China; Department of Integrated TCM & Western Medicine, Jiangsu Cancer Hospital, Jiangsu Institute of Cancer Research & The Affiliated Cancer Hospital of Nanjing Medical University Nanjing Jiang Su 210000 China 41186709@qq.com; Department of Medicine, Jiangsu Cancer Hospital, Jiangsu Institute of Cancer Research & The Affiliated Cancer Hospital of Nanjing Medical University Nanjing Jiang Su 210000 China

## Abstract

Correction for ‘A microRNA-4516 inhibitor sensitizes chemo-resistant gastric cancer cells to chemotherapy by upregulating ING4’ by Jun-bao Liu *et al.*, *RSC Adv.*, 2018, **8**, 37795–37803.

Errors were present in the published article in terms of corresponding authors and e-mail addresses; Chen Yu is the sole corresponding author, extra e-mail addresses have been deleted, and the correct version is shown here.

The Royal Society of Chemistry apologises for these errors and any consequent inconvenience to authors and readers.

## Supplementary Material

